# Digital adherence technology to improve medication adherence in tuberculosis patients: a systematic review and meta-analysis randomized control trials

**DOI:** 10.1038/s41533-025-00457-3

**Published:** 2025-11-21

**Authors:** Agus Santosa, Neti Juniarti, Tuti Pahria, Raini Diah Susanti

**Affiliations:** 1 Nursing Doctoral Program, Faculty of Nursing, Universitas Padjadjaran, Sumedang, West Java Indonesia; 2 Department of Nursing, Faculty of Health Sciences, Universitas Muhammadiyah Purwokerto, Banyumas, Central Java Indonesia; 3 Department of Community Health Nursing, Faculty of Nursing, Universitas Padjadjaran, Sumedang, West Java Indonesia; 4 Department of Medical-Surgical Nursing, Faculty of Nursing, Universitas Padjadjaran, Sumedang, West Java Indonesia

**Keywords:** Health care, Health policy

## Abstract

Medication adherence is critical for successful tuberculosis (TB) treatment, yet non-adherence remains a major barrier to TB control globally. Digital adherence technologies (DAT) have emerged as promising tools to support adherence, but their effectiveness remains variably reported across settings and intervention types. To evaluate the effectiveness of DAT compared to directly observed therapy (DOT) in improving TB medication adherence through a systematic review and meta-analysis of randomized controlled trials (RCTs). A comprehensive literature search was conducted across PubMed, Scopus, EBSCO, and ScienceDirect from inception through November 7, 2024. RCTs comparing DAT (e.g., SMS reminders, video-observed therapy [VOT], medication event reminder monitors [MERM], biometric monitoring systems [BMS], ingestion sensors [IS]) with DOT were included. Study selection, data extraction, and quality appraisal were performed independently by multiple reviewers. Meta-analyses were conducted using a random-effects model, with subgroup and sensitivity analyses. This review followed the PRISMA 2020 reporting guidelines. Nineteen RCTs involving over 10,000 TB patients were included. Overall, DAT significantly improved medication adherence compared to DOT, with a pooled odds ratio (OR) of 2.853 (95% CI: 2.144–3.796; *p* < 0.001). Subgroup analyses indicated that VOT, MERM, and SMS reminder were consistently effective, while the highest effect sizes were seen in IS and BMS, albeit with wider confidence intervals. Effectiveness varied by country income level: DAT were more effective in high- and upper-middle-income countries, while findings in lower-income settings remained inconclusive, partly due to the limited number of studies. Sensitivity analysis confirmed the robustness of findings, and no significant publication bias was detected (Egger’s test *p* = 0.979). DAT are significantly more effective than DOT in improving medication adherence among TB patients. Tailored implementation strategies are needed to ensure optimal selection and integration of DATs across diverse health systems. These findings support the scaling-up of context-appropriate digital tools as part of global TB control efforts.

## Introduction

Tuberculosis (TB) remains a significant global health challenge, with an estimated 10.8 million new cases and 1.25 million deaths reported in 2023^1^. Despite advancements in treatment and prevention strategies, TB continues to disproportionately affect low- and middle-income countries, where socioeconomic barriers and limited healthcare access exacerbate its burden^2^.

Adherence to TB treatment is critical for achieving positive patient outcomes, preventing drug resistance, and reducing community transmission^3,4^. In addition to side effects—which are commonly reported by TB patients—the prolonged duration of TB treatment often leads to forgetfulness and treatment fatigue^5–7^. These factors further complicate adherence and underscore the importance of sustained engagement throughout the course of therapy.

Directly observed therapy (DOT) has long been the standard approach to ensuring adherence, offering supervised medication intake—whether by a family member, health worker, or community-based provider—to improve outcomes^8^. While DOT has demonstrated efficacy, its implementation faces significant challenges, including limited family support, insufficient staffing, and concerns regarding patient acceptability^9–11^.

In response to these limitations, digital adherence technologies (DAT) have emerged as innovative, patient-centered alternatives. These tools include short message service (SMS) reminders^12,13^, video-observed therapy (VOT)^14,15^, digital pillboxes^16,17^, and ingestible sensors^18^, all of which provide scalable, customizable solutions to support patients throughout their treatment journeys.

Despite the growing adoption of DAT, evidence of their effectiveness is inconsistent. SMS reminder have shown mixed results, with some studies reporting significant improvements in adherence rates while others find negligible effects^12,13^. Similarly, VOT has demonstrated variability in effectiveness depending on the context^14,15^. Digital pillboxes, while promising, are hindered by high implementation costs^16,17^, and ingestion sensors raise concerns about patient acceptance due to potential long-term side effects and other factors^19^.

Although several systematic reviews have investigated the effectiveness of individual DAT such as VOT and SMS reminders in TB treatment, they exhibit notable limitations in scope and methodological rigor. Sundaram et al. focused exclusively on VOT, relying largely on cohort studies and offering limited generalizability due to the absence of randomized controlled trials (RCTs)^20^. Similarly, Nglazi et al. examined SMS interventions but identified only one RCTs, with overall low-quality evidence and high heterogeneity^21^. Sholikhah et al. included various digital health interventions, yet lacked a clear framework for evaluating adherence outcomes and did not exclusively focus on RCTs^22^.

Ridho et al. provided a broader synthesis of multiple DAT types using RCTs, but did not perform subgroup meta-analyses by intervention type or country income level, leaving critical contextual variations unexplored^23^. Collectively, these limitations underscore the need for a comprehensive and methodologically robust systematic review and meta-analysis that focuses exclusively on RCTs, incorporates subgroup comparisons, and provides nuanced insights into the differential effectiveness of DAT across settings and technologies.

This systematic review and meta-analysis aims to synthesize evidence from RCTs on the effectiveness of various DAT in improving TB medication adherence. Specifically, the study compares DAT effectiveness across different country income levels and intervention types, including comparisons with traditional DOT approaches. Findings from this review are expected to inform future policy, guide clinical practice, and support the integration of appropriate digital solutions into national TB programs worldwide.

## Methods

### Protocol and registration

This systematic review and meta-analysis was conducted in accordance with the preferred reporting Items for systematic reviews and meta-Analyses (PRISMA) guidelines. The review protocol was registered with the international prospective register of systematic reviews (PROSPERO) under the registration number CRD42024629655 (available at: https://www.crd.york.ac.uk/prospero/display_record.php?ID=CRD42024629655).

### Eligibility criteria

In this systematic review, the inclusion criteria focused on RCTs involving patients diagnosed with tuberculosis who received interventions utilizing DAT (such as SMS reminder, VOT, digital pillboxes (e.g., MERM), *etc*.) compared to those undergoing DOT, including observation by health workers, family members, or community volunteers to improve medication adherence. Studies were required to report on outcomes related to medication adherence rates among tuberculosis patients. Studies were included if they reported medication adherence outcomes in a quantifiable manner (e.g., treatment completion, missed doses, cure rate), regardless of whether adherence was the primary or secondary outcome. Conversely, studies that were excluded from this review included observational studies (including cohort, cross-sectional, and case-control designs), systematic reviews, meta-analyses, editorials, and study protocols that were not considered for inclusion.

### Information sources and search strategy

A comprehensive literature search was conducted across four major databases: PubMed, Scopus, EBSCO (Medline), and ScienceDirect, from inception to November 7, 2024. These databases were selected due to their comprehensive coverage of biomedical literature and frequent use in prior systematic reviews on similar topics. The search strategy combined MeSH terms and free-text keywords related to tuberculosis, digital adherence technologies, and treatment adherence. The search was tailored to each database using Boolean operators and truncations to optimize sensitivity. The search strategy followed the PICO framework, where: P (Population): patients with tuberculosis; I (Intervention): digital adherence technologies (e.g., SMS, VOT, MERM); C (Comparison): directly observed therapy; O (Outcome): medication adherence. The full search strings for each database are provided in Appendix A.

### Data collection process and data items

Data extracted from each included study comprised the first author’s name and year of publication, country of study along with its world bank income classification (2024), study design and setting, sample size, and participant characteristics. Information on the intervention, including the type of digital adherence technology employed (e.g., SMS reminder, video-observed therapy), and the comparator intervention (typically directly observed therapy), was also recorded. Additionally, primary and secondary outcome measures related to medication adherence, treatment completion, and treatment success were documented.

Medication adherence was defined based on the original study definitions, which varied across trials and included metrics such as treatment completion, missed doses, cure rates, and validated adherence scales. In this review, adherence was primarily defined based on direct measures (e.g., missed doses). However, when such data were unavailable, surrogate indicators such cure rate were considered proxy measures for adherence, following common practices in TB adherence research. For meta-analysis, all outcomes were dichotomized into adherence versus non-adherence to calculate effect sizes.

All authors independently screened titles and abstracts for initial eligibility. Subsequently, full-text articles were assessed against the predefined inclusion criteria. Any discrepancies during the screening or data extraction processes were resolved through discussion and consensus among all reviewers.

### Risk of bias assessment

The methodological quality of included RCTs was assessed using the JBI critical appraisal checklist for randomized controlled trials^24^. Studies were rated across 13 criteria, yielding quality scores categorized as follows: high quality: 9–13; moderate quality: 5–8; low quality: 1–4. All authors independently conducted the appraisal. Disagreements were resolved through consensus. Three independent reviewers conducted the appraisal process. Disagreements were resolved through discussion and consensus to ensure reliability. The purpose of the quality assessment was to evaluate the internal validity and methodological rigor of included studies, thereby ensuring that synthesized results were based on robust and credible evidence.

### Data synthesis and statistical analysis

A meta-analysis was performed to estimate the pooled effect size of DAT on medication adherence compared to DOT. The results were synthesized using a random-effects model, considering variability across studies. Effect sizes were presented as OR with 95% CI, based on raw dichotomous data extracted from each study (i.e., number of adherent patients and total number per group). These calculations were performed using comprehensive meta-analysis (CMA) software, which enabled standardization across studies regardless of whether OR were originally reported.

Forest plots were used to visualize individual and pooled effect sizes. Subgroup analyses were conducted by type of DAT and country income level. Heterogeneity was assessed using the I² statistic, with values above 50% indicating substantial heterogeneity. Publication bias was evaluated using funnel plots and Egger’s regression test.

## Results

### Study selection

**Fig. 1:**
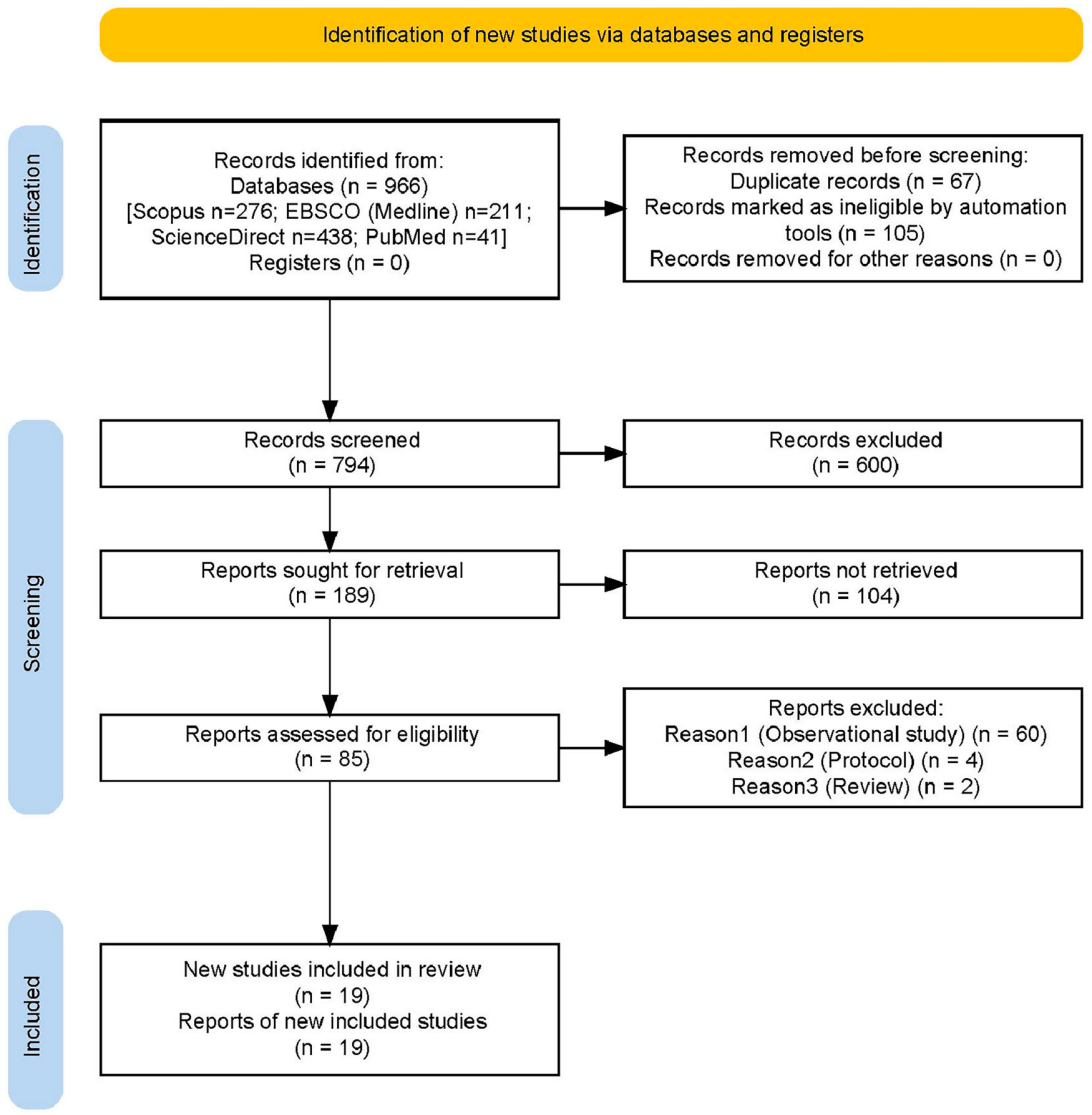
PRISMA 2020 flow diagram illustrating the study selection process.

The study selection process followed the PRISMA 2020 guidelines and is summarized in Fig. 1. A total of 966 records were retrieved from four major databases. After removing 67 duplicates, 105 additional records were marked as ineligible by automated reference management tools due to incomplete metadata or import errors, 794 unique records were screened. Following title and abstract screening, 600 were excluded. Of the remaining 189 reports sought for retrieval, 104 were excluded after full-text screening because they due to evaluating only DAT or DOT without a comparator group. A total of 85 full-text articles were assessed for eligibility, resulting in the exclusion of 60 observational studies, 4 study protocols, and 2 systematic reviews. Ultimately, 19 randomized controlled trials met the inclusion criteria and were included in the final synthesis. A more detailed breakdown of search results by database and filtering process is provided in Appendix B.

### Characteristics of studies

This review included 19 RCTs published between 2015 and 2024, all of which evaluated the effectiveness of various DAT in improving tuberculosis treatment outcomes compared to conventional DOT. The studies were conducted in 13 countries, including Cameroon (n = 1)^12^, China (n = 5)^25–29^, Ethiopia (n = 2)^17,30^, Kenya (n = 1)^31^, Moldova (n = 1)^14^, South Africa (n = 1)^32^, Sudan (n = 1)^33^, Thailand (n = 1)^34^, Uganda (n = 1)^35^, the United Kingdom (n = 1)^36^, and the United States (n = 4)^15,37–39^. Sample sizes ranged from 61 to 4173 participants, and most studies were multi-site or cluster-randomized trials conducted in real-world TB care settings.

Across these studies, SMS reminder were the most frequently evaluated DAT, employed in seven studies^12,25,26,29–31,33^. VOT was evaluated in eight studies^12,14,15,27,28,34,36,37^, showing consistent evidence of improved adherence and patient acceptability. Three studies investigated the use of MERM^17,25,32^, while another examined biometric monitoring systems (BMS)^35^ and One study employed ingestion sensors (IS) with wirelessly observed therapy to confirm dose intake^38^. In addition, one study trials implemented multi-component interventions combining more than one DAT approach, including those by Liu et al.^25^, which integrated SMS reminder with electronic monitoring.

Outcomes measured included medication adherence, treatment completion, number of missed doses, and cure rates. Intervention arms using DAT reported adherence rates ranging from 79 to 99%, compared with 61.5 to 86.8% in DOT control groups. Notably, studies using MERM and SMS combinations demonstrated significant reductions in missed doses^25,29^, while video-based interventions consistently improved adherence and patient satisfaction^27,36,37^. Collectively, these trials involved over 10,000 patients and provide strong comparative evidence supporting the integration of DAT into TB programs. A summary of study characteristics is presented in Table 1.

**Table 1:** Characteristics of studies (n = 19).

No	Author	Year	Country and income level	Study Design	Sample Size	Intervention Details	Adherence Measurement	Key Outcomes
1.	Liu et al.^25^	2015	China, Upper-middle-income	A Cluster-Randomised Trial	•Total sample size: 4173 participants •Sample size for the intervention Group: Text messaging group: 1008 participants. Medication monitor group: 997 participants; Combined group (Text Messaging + Medication Monitor): 1064 participants •Sample size for the control group: 1104 participants	Compared three intervention strategies: text messaging, medication monitor box, and combined against DOT (supervised by family members or health care workers).	Missed dose	The percentage of patient-months with at least 20% doses missed was 29.9% (control), 27.3% (SMS), 17.0% (monitor), and 13.9% (combined); significant reduction in poor adherence in monitor and combined arms (aMR 0.58, p < 0.01).
2.	Snidal et al.^35^	2015	Uganda, Lower-middle-income	Pilot study controlled	•Total sample size: participants •Sample size for the intervention group: 142 participants (eCompliance system) •Sample size for the control group: 106 participants (community-based DOT)	Implemented a biometric monitoring system (eCompliance) fingerprint verification to enhance adherence tracking compare daily supervision by a family member.	Cure rate	The cured rate was 55.6% in the intervention group vs. 28.3% in the control (p < 0.01); loss to follow-up was 0% in the intervention vs. 7% in the control (p < 0.05).
3.	Fang et al.^26^	2017	China, Upper-middle-income	A prospective, randomized controlled study	•Total sample size: 350 participants •Sample size for the intervention group (SMS): 160 participants •Sample size for the control group (DOT): 190 participants	SMS reminder for treatment adherence were compared against traditional DOT.	Completion rate	The treatment completion rate was 96.25% in the SMS group vs. 86.84% in the control group (p = 0.002); reducing the missed dose rate and interrupted treatment rate significantly (p < 0.001).
4.	Garfein et al.^37^	2018	USA, High-income	Prospective, multisite, single-arm control trial	•Total sample size: 433 participants •Sample size for the intervention group (VDOT): 274 participants •Sample size for the control group (DOT): 159 participants	Compared VDOT with in-person DOT.	Median fraction of expected doses observed	The median fraction of expected doses observed was 93.0% for VDOT vs. 66.4% for DOT (p < 0.001); 96% of participants preferred VDOT over DOT.
5.	Bediang et al.^12^	2018	Cameroon, Lower-middle-income	A simple blinded, randomized controlled trial	•Total sample size: 279 •Sample size for the intervention Group (DOT + SMS): 137 •Sample size for the control group: 142	Patients in the intervention group received daily SMS reminder in addition to standard DOT; the control group received only standard DOT.	Cure rate	Treatment success at 5 months: 81% (intervention) vs 74.6% (control) p = 0.203; Cure rate at 6 months: 63.5% (intervention) vs 62% (control) p = 0.203. Dropout rates were similar, and no significant differences were observed in secondary outcomes such as patient adherence and satisfaction.
6.	Ali and Prins.^33^	2019	Sudan, Lower-middle-income	Pilot study controlled	•Total sample size: 148 participants •Sample size for the intervention group: 74 participants (received SMS reminders) •Sample size for the control group: 74 participants (did not receive SMS reminders)	SMS reminder compared with standard care (DOTS).	Cure rate	The default rate was 6.8% in the intervention group vs. 10.8% in the control (p = 0.563); the cure rate was 78.4% in intervention vs. 59.5% in control (p = 0.020).
7.	Story et al.^36^	2019	England, High-income	A multicentre, analyst-blinded, randomized controlled, superiority trial	•Total sample size: 226 participants •Sample size for the intervention group (VOT): 112 participants •Sample size for the control group (DOT): 114 participants	Compare smartphone-enabled video-observed therapy (VOT) with directly observed therapy (DOT) by a healthcare or lay worker, with the remaining daily doses self-administered.	Missed dose observation	In Intention-to-Treat (ITT) analysis, 78 (70%) of VOT patients achieved ≥80% scheduled observations compared to 35 (31%) of DOT patients (p < 0.0001); VOT demonstrated a higher completion rate and fewer adverse events.
8.	Browne et al.^38^	2019	USA, High-income	Randomized controlled trial	•Total sample size: 61 participants •Sample size for the intervention group (Ingestion sensor): 41 participants •Sample size for the control group (DOT): 20 participants	Compared wirelessly observed therapy using an ingestion sensor with DOT.	Missed dose observation	Ingestion sensor group confirmed 93% of prescribed doses compared to 63% for DOT (p < 0.001); 100% of participants preferred WOT; adverse events were low ( < 10%).
9.	Ravenscroft et al.^14^	2020	Moldova, Upper-middle-income	2-arm individually randomized clinical trial	•Total sample size: 197 participants •Sample size for the intervention group (VOT): 98 participants •Sample size for the control group (DOT): 99 participants	Compared asynchronous VOT with in-person DOT.	Missed dose	•Adherence rate for intervention group (VOT): 90.7% (median number of days missed per two-week period was 1.29 days) •Adherence rate for control group (DOT): 62.6% (5.24 days missed per two-week period) •Did not find a significant impact of treatment allocation on treatment success by sputum smear and X-Ray at 4 months after starting the continuations phase (DOT = 15.0%, VOT = 11.1%), 10 months (DOT = 86.0%, VOT = 92.1%), or 12 months (DOT = 90.3%, VOT = 93.5%)
10.	Guo et al.^27^	2020	China, Upper-middle-income	Randomized controlled trial	•Total sample size: 405 •Sample size for the intervention group (VDOT): 203 •Sample size for the control group (DOT): 202	Video directly observed therapy (VDOT) vs directly observed therapy (DOT)	Completion rate	Treatment completion rates: 96.1% (VDOT) vs. 94.6% (DOT) had no statistical differences; cure rate 91,6% (VDOT) vs. 87,6% (DOT) p = 0.187; Time per dose observed: 16.5 min (VDOT) vs. 44.1 min (DOT), VDOT group had better patient satisfaction and acceptance, Cost-effective: VDOT cost was significantly lower than DOT.
11.	Gashu et al.^30^	2021	Ethiopia, Low-income	Randomized controlled trial	•Total sample size: 306 •Sample size for the intervention group: 152 •Sample size for the control group: 154	Phone-based reminder system for weekly pill refilling and daily medication reminders via text and graphics compare routine care (community health worker, family member, neighbour, workmate, or community figure trained to observe)	Medication adherence scale	Adherence to TB treatment: 79.1% (intervention) vs. 66.4% (control), RR = 1.632 (p = 0.018)
12.	Manyazewal et al.^17^	2022	Ethiopia, Low-income	A multicenter randomized controlled trial	•Total sample size: 114 participants •Sample size for the intervention group: 57 participants (using the digital medication event reminder) •Sample size for the control group: 57 participants (standard care)	Compared the medication event reminder and monitor (MERM) device for self-administered therapy (The evriMED500 dispenser consists of a medication container and an electronic module) against traditional DOT (by healthcare provider).	Missed dose	Adherence was non-inferior: MERM group had 99.01% adherence vs. 98.97% for DOT (MR 1.00; p = 0.954).
13.	Burzynski et al.^39^	2022	USA, High-income	A randomized, 2-arm, 2-period crossover, noninferiority design	•Total sample size: 173 participants •Sample size for the intervention group (Electronic DOT): 173 participants •Sample size for the control group (In-Person DOT): 173 participants	Compared electronic DOT (live video conferencing or recorded videos) with in-person DOT (clinic or community-based DOT).	Completed dose	Completion rates were 89.8% for electronic DOT vs. 87.2% for in-person DOT (difference −2.6%, 95% CI −4.8% to −0.3%); demonstrated noninferiority of electronic DOT.
14.	Boutilier et al.^31^	2022	Kenya, Lower-middle-income	Randomized controlled trial	•Total sample size: 1046 participants •Sample size for the intervention group: 541 participants •Sample size for the control group: 505 participants	Evaluated a novel DAT-based behavioral support program, including SMS reminder and adherence verification via USSD, compared with the standard of care (DOT)	Success rate	•Adherence rate for intervention group: 0.044 (or 4.4% unsuccessful treatment outcome) •Adherence rate for control group: 0.128 (or 12.8% unsuccessful treatment outcome)
15.	Liu et al.^29^	2023	China, Upper-middle-income	Cluster-randomized superiority trial	•Total sample size: 2686 participants (of the 3074 enrolled) •Sample size for the intervention Group: 1298 participants •Sample size for the control group: 1388 participants	A daily reminder monitor and monthly adherence review compare DOT (observation by a healthcare worker or family member)	Missed dose	•Months in which patient missed >20% of doses per person per month of treatment intervention group 0·9/6·0 (16%), control group 2·7/6·0 (46%) •Doses missed per person per doses expected intervention group 16/160 (11%), control group 42/160 (27%) •Late or missed clinic visits per person per scheduled visits intervention group 2·5/5·0 (49%), control group 2·6/5·0 (51%) •Reduced non-adherence by 57–64% in the intervention group compared to control (p < 0.001).
16.	Guo et al.^28^	2023	China, Upper-middle-income	Randomized controlled trial	•Total sample size: 167 participants •Sample size for the intervention group (eDOTS): 93 participants (43 from villages and 50 from city neighborhoods) •Sample size for the control group (DOTS): 74 participants (38 from villages and 36 from city neighborhoods)	Electronic DOTS (eDOTS) is an electronic medicine box (eBox) for patients, a private mobile phone (management software program I, WeChat App installed, for all of the participants, including persons with active TB, doctors, nurses, management Staff compared with traditional DOTS in managing treatment adherence.	Missed dose	•Adherence rate for intervention group (eDOTS) in Village: 49.8% ± 26.9% (during the first week) and 77.8% ± 17.1% (after follow-up calls at week 21) •Adherence rate for intervention group (eDOTS) in city neighborhoods (98.9% ± 1.1%; t = −32.781, p < 0.001) •After 6 months of treatment, the average treatment compliance of the village patients who used eDOTS was 47.0% ± 20.5% compared to 26.7% ± 21.1% who used DOTS (t = −4.475, p < 0.001)
17.	Charalambous et al.^32^	2024	South Africa, Upper-middle-income	A cluster randomized trial	•Total sample size: 2584 participants adherence data •Sample size for the intervention Group: 1306 participants •Sample size for the control group (standard of care): 1278 participants	Participants in the intervention arm received a differentiated care adherence package (Wisepill EvriMED medication monitor, SMS reminder, and follow-ups (phone calls and home visits)) versus standard of care (SoC) DOT.	Missed dose	Adherence ≥80% was significantly higher in the intervention (81.0%; 1056 out of 1306) versus SoC (50.8%; 650 out of 1278), with an adjusted risk ratio of 1.51 (95% CI 1.36–1.66); overall adherence was 88.5% in intervention vs. 69.7% in SoC (p < 0.001).
18.	Kumwichar et al.^34^	2024	Thailand, Upper-middle-income	Cluster Randomized Controlled Trial	•Total sample size: 128 participants •Sample size for the intervention group (VDOT): 63 participants •Sample size for the control group (DOT): 65 participants	Compare smartphone-based VOT with community-based DOT.	Missed dose	•VOT showed a higher average compliance of 67.5 days vs. 51.2 days for DOT (p = 0.005); •Patient compliance rate in the VOT group approached 45.13% (1706/3780) over the 60-day. In contrast, the patient compliance rate in the DOT group was 20.9% (815/3900) over the 60 days •The sputum conversion rates in the VOT and DOT groups were 73% and 61.5%, respectively (p = 0.17)
19.	Garfein et al.^15^	2024	USA, High-income	Randomized controlled trial	•Total sample size: 130 participants •Sample size for the intervention group (VDOT): 68 participants •Sample size for the control group (DOT): 62 participants	Compared VDOT (self-recorded videos of medication ingestion) with clinic-based DOT.	Completion rate	Treatment completion was 73.5% for VDOT vs. 69.4% for DOT (p = 0.70)

Exp: Mean Ratio (MR); Adjusted Mean Ratio (AMR); DOT and DOTS refer to in-person directly observed therapy; VOT, VDOT, and eDOTS refer to video-observed therapy.

### The result of quality assessment and publication bias

**Fig. 2:**
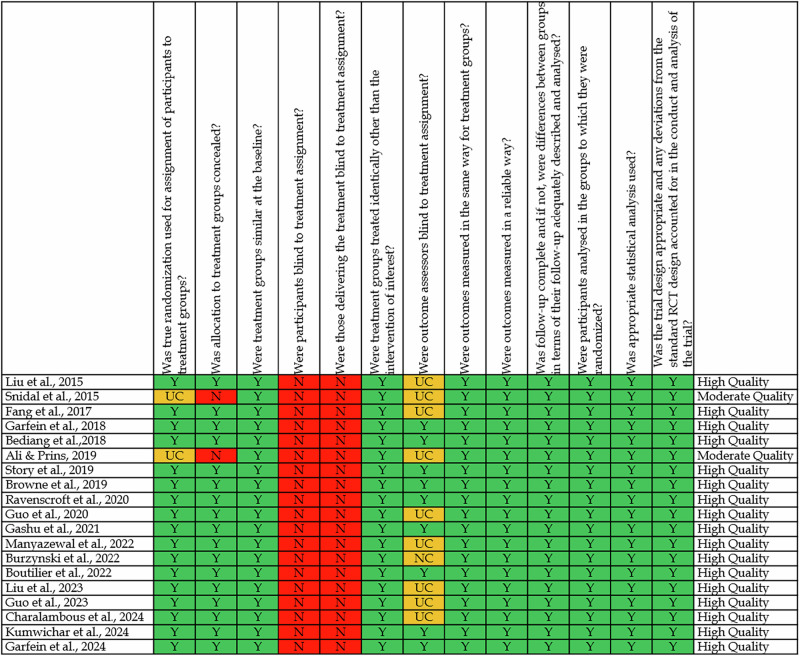
Risk of bias assessment using the JBI critical appraisal checklist for RCTs.

The methodological quality of the 19 included RCTs was evaluated using the JBI critical appraisal checklist. As shown in Fig. 2, 15 studies were rated as high quality, and 2 as moderate^33,35^, while two studies presented unclear risks due to limited reporting on blinding and allocation. Most trials adequately reported randomization methods, baseline comparability, and outcome measurement.

A consistent limitation across studies was the absence of blinding, especially among participants and personnel, which is understandable given the nature of interventions like SMS or VOT. Outcome assessor blinding was also not reported in several studies (e.g., Fang et al.; Bediang et al.)^12,26^, raising potential detection bias. Nonetheless, most studies maintained complete follow-up and used appropriate statistical analysis. These findings indicate an overall low-to-moderate risk of bias, with strong internal validity for most included studies, although limitations in blinding should be taken into account.

### Main findings

**Fig. 3:**
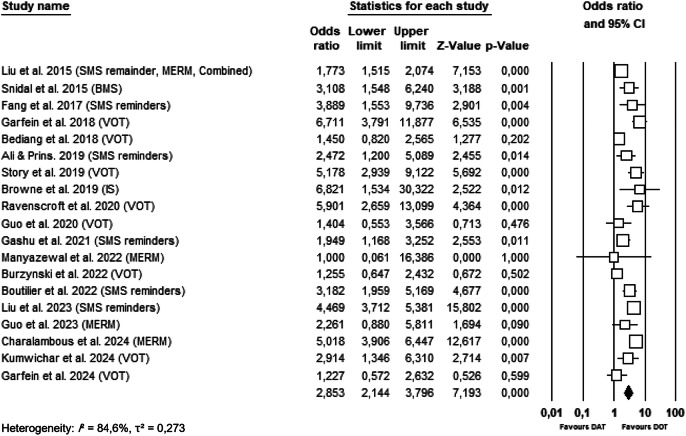
Forest plot of the effectiveness of DAT versus DOT on treatment adherence in tuberculosis patients.

The meta-analysis included 19 randomized controlled trials assessing the effectiveness of DAT in improving medication adherence among tuberculosis patients, compared to DOT. As illustrated in Fig. 3, the overall pooled OR for medication adherence in the DAT intervention groups compared to control groups was 2.853 (95% CI: 2.144–3.796; *p* < 0.001). This finding indicates that patients who received DAT-based interventions were nearly three times more likely to adhere to their TB treatment regimen than those receiving standard DOT.

The forest plot demonstrates that most individual studies reported statistically significant improvements in adherence, with several studies showing OR above 2.0 and narrow confidence intervals (e.g., Liu et al.; Boutilier et al.; Guo et al.)^28,29,31^. Some of the most effective interventions included combinations of SMS reminder and MERM devices^25^, as well as VOT approaches^36,37^.

However, a few studies showed wide confidence intervals or non-significant findings, particularly in trials with smaller sample sizes or those testing early-stage technologies, such as ingestion sensors^38^ and electronic VOT systems^39^. For instance, Guo et al.^27^ reported an OR of 1.404 (95% CI: 0.553–3.566), and Garfein et al.^15^ found no statistically significant difference between groups (OR = 1.227; 95% CI: 0.572–2.632). Due to the substantial heterogeneity observed across studies (I² = 84.6%, *p* < 0.001), a random-effects model was used in the meta-analysis.

### Subgroup analyses by type of DAT

**Fig. 4:**
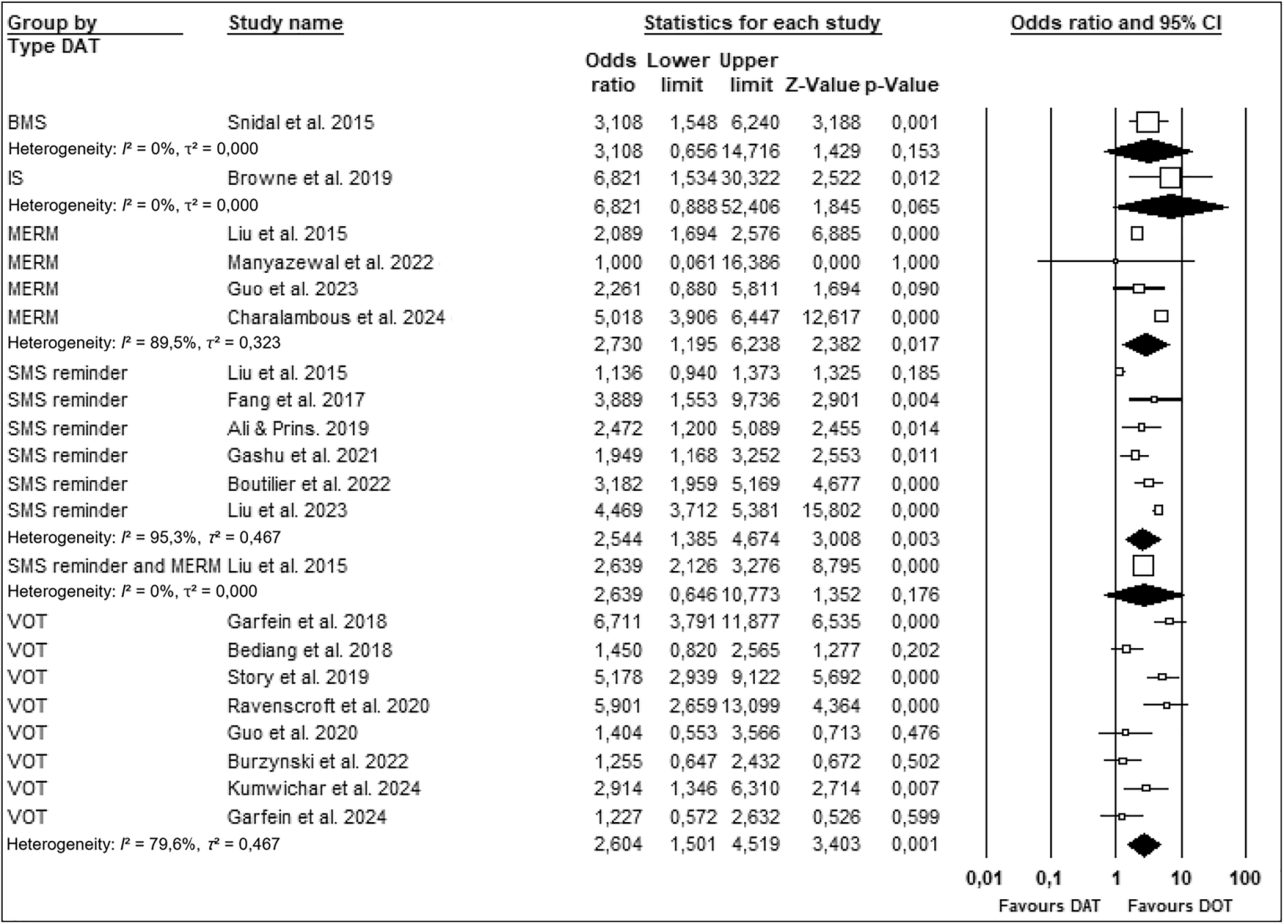
Forest plot of subgroup analysis based on type of DAT.

Figure 4 demonstrate that while all DAT categories showed a positive trend in improving tuberculosis medication adherence, the magnitude and statistical significance varied across intervention types. Subgroup analyses were conducted using a random-effects model to account for expected clinical and methodological heterogeneity across studies, even when statistical heterogeneity (I²) was low. This approach improves generalizability across diverse settings and patient populations.

Results showed that VOT (8 studies) significantly improved medication adherence (OR: 2.604; 95% CI: 1.501–4.519; *p* = 0.001)^12,14,15,27,34,36,37,39^, as did SMS reminder (6 studies; OR: 2.544; 95% CI: 1.385–4.674; *p* = 0.003)^25,26,29–31,33^ and MERM (4 studies; OR: 2.730; 95% CI: 1.195–6.238; *p* = 0.017)^17,25,28,32^. These findings were consistent and robust across multiple trials. In contrast, single-study subgroups such as BMS (OR: 3.108; 95% CI: 0.656–14.716; *p* = 0.153)^35^, ingestion sensors (OR: 6.821; 95% CI: 0.888–52.406; *p* = 0.065)^38^, and combined SMS + MERM (OR: 2.636; 95% CI: 0.646–10.773; *p* = 0.176)^25^ showed high effect sizes but non-significant results and wide confidence intervals, indicating the need for further research. Collectively, the evidence suggests that VOT, SMS, and MERM are the most consistently effective DATs currently supported by the literature.

Subgroup analyses by type of DAT revealed substantial heterogeneity across several subgroups. I² values indicated high between-study variability in the MERM (I² = 89.5%), SMS reminder (I² = 95.3%), and VOT (I² = 79.6%) groups, suggesting that the effect sizes varied considerably among included studies within these subgroups. These values were derived from fixed-effects heterogeneity statistics, as the CMA software does not directly report I² under the random-effects model.

### Subgroup analyses by country income level

**Fig. 5:**
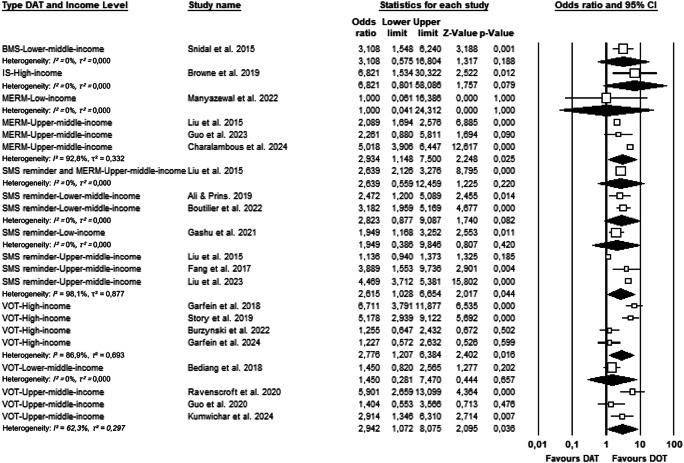
Forest plot of subgroup analysis based on country income level.

Figure 5 reveal variation in the effectiveness of DAT across different economic settings. In high-income economies, two types of DAT were assessed. IS showed a high point estimate (OR: 6.821; 95% CI: 0.801–30.320; *p* = 0.079)^38^, but the wide confidence interval and non-significant p-value suggest limited precision, likely due to a small sample size. In contrast, VOT, analyzed in multiple studies^15,36^, demonstrated a statistically significant effect (OR: 2.776; 95% CI: 1.207–6.384; p = 0.016), indicating robust support for its use in high-income settings.

In upper-middle-income economies, several DAT demonstrated statistically significant benefits. MERM^25,28,32^ showed a pooled OR of 2.934 (95% CI: 1.480–7.500; *p* = 0.025), while SMS reminder^25,26,29^ yielded an OR of 2.615 (95% CI: 1.026–6.654; *p* = 0.044). VOT also widely studied in this group, showed a pooled OR of 2.942 (95% CI: 1.072–8.075; *p* = 0.036)^15,36,37,39^. Interestingly, a combined intervention of SMS reminder and MERM showed a strong point estimate (OR: 2.639), but with a wide confidence interval and a non-significant p-value (95% CI: 0.559–12.459; *p* = 0.220)^25^.

In lower-middle-income economies, the effectiveness of DAT appeared more variable. BMS showed a favorable but non-significant effect (OR: 3.108; 95% CI: 0.575–16.804; *p* = 0.188), as did SMS reminder (OR: 2.823; 95% CI: 0.877–9.087; p = 0.082) and VOT (OR: 1.450; 95% CI: 0.281–7.470; *p* = 0.657)^12,31,33,35^. The wide confidence intervals and non-significant p-values suggest greater uncertainty in these settings, potentially due to contextual or operational challenges in implementation.

In low-income economies, two studies assessed MERM and SMS reminder. MERM^17^ showed no effect (OR: 1.000; 95% CI: 0.041–24.312; *p* = 1.000), while SMS reminder^30^ yielded an OR of 1.949 (95% CI: 0.386–9.846; *p* = 0.420), both of which were not statistically significant. These findings suggest that DAT appear to be more consistently effective in upper-middle and high-income settings, while results in lower-income contexts remain inconclusive and warrant further investigation, particularly to understand barriers related to infrastructure, user literacy, and system readiness.

While the analysis provides useful directional insights into how income level may influence the effectiveness of DATs, the limited number of studies in several subgroups—particularly in low-income and lower-middle-income contexts—warrants cautious interpretation. These results are intended to guide hypothesis generation and highlight the need for more research in underrepresented settings.

Subgroup analysis based on country income level revealed considerable heterogeneity among some categories. Notably, high heterogeneity was observed in MERM interventions conducted in upper-middle income countries (I² = 92.8%), SMS-based interventions (I² = 98.1%), VOT in high-income countries (I² = 86.9%), and VOT in upper-middle income countries (I² = 62.3%). These findings suggest substantial variation in effect sizes depending on the economic context and the type of digital adherence technology. For other subgroups with only one included study, heterogeneity estimates (I²) were not applicable.

### Sensitivity analysis

**Fig. 6:**
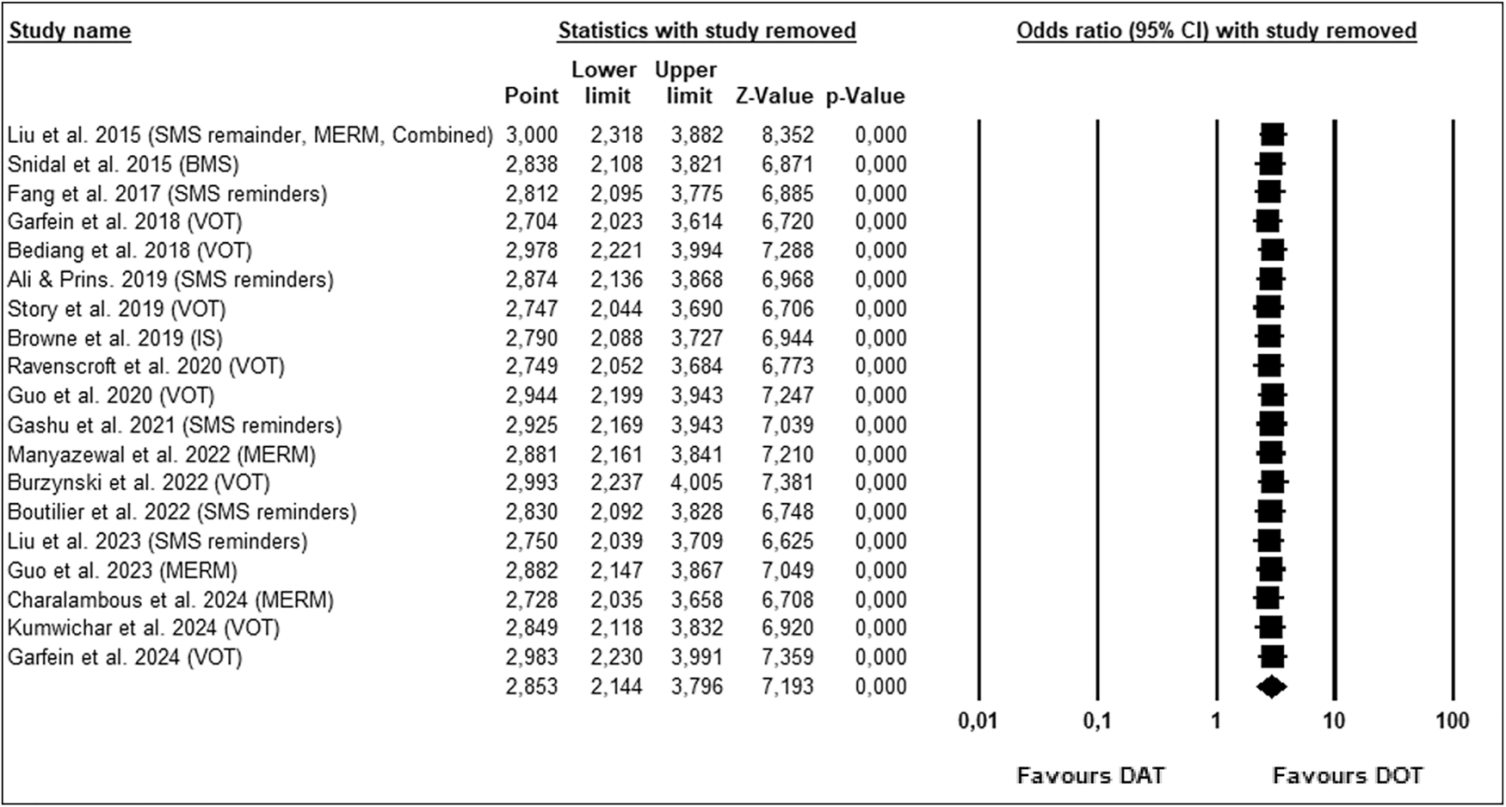
Leave-one-out sensitivity analysis.

A leave-one-out sensitivity analysis was conducted to evaluate the robustness of the pooled effect estimate. Each of the 19 included studies was sequentially removed, and the meta-analysis was re-run to assess whether any single study had a disproportionate influence on the overall results. The pooled odds ratios across all iterations ranged from 2.704 to 3.000, and the results remained statistically significant in all cases (*p* < 0.001). These findings indicate that the meta-analysis is robust and not unduly influenced by any individual study. The stability of the results strengthens confidence in the conclusion that digital adherence technologies significantly improve tuberculosis medication adherence compared to DOT (Fig. 6).

### Publication bias

**Fig. 7:**
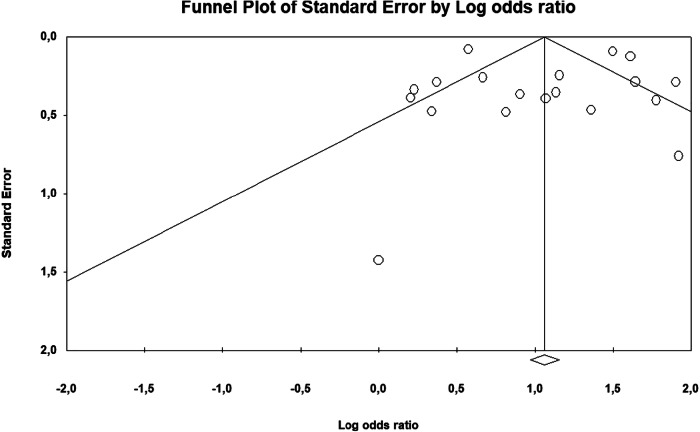
Funnel plot of standard error by log odds ratio. While the plot shows slight asymmetry, Egger’s regression test did not indicate significant publication bias (*p* = 0.979).

Publication bias was evaluated through both visual inspection of the funnel plot and Egger’s regression asymmetry test. While the funnel plot (Fig. 7) showed some degree of visual asymmetry, which may be influenced by the distribution of small studies, this was not statistically confirmed. Egger’s test yielded a non-significant result (*p* = 0.979), indicating no evidence of small-study effects or publication bias. Taken together, these findings suggest a low risk of publication bias and support the robustness of the meta-analysis findings.

## Discussion

This study aimed to synthesize evidence from RCTs evaluating the effectiveness of DAT in improving TB medication adherence. The findings provide robust support for the integration of DAT into TB treatment programs. The pooled analysis of 19 RCTs showed a significant improvement in adherence among patients using DAT compared to those receiving DOT, with a pooled OR of 2.85 (95% CI: 2.14–3.80; *p* < 0.001). These results reinforce the value of DAT as scalable, patient-centered interventions for enhancing treatment outcomes in TB care.

Our findings align with prior reviews that underscore the potential of mobile health interventions in TB management. Systematic reviews by Sholikhah et al.^22^, Ridho et al.^23^, and Nglazi et al.^21^ reported that tools such as SMS reminder, electronic pillboxes, and video-based technologies can enhance adherence across both TB and other chronic disease populations^21–23^. However, this study advances the literature by including recently published RCTs through 2024 and disaggregating the effects of DAT by both intervention type and country income level. This stratified approach allows for a more nuanced interpretation of what works, for whom, and under what conditions.

Subgroup analyses revealed meaningful variation in effectiveness across DAT types. IS and BMS demonstrated high effect estimates, but these results were drawn from single studies. Therefore, they should be interpreted as individual outcomes rather than pooled effects, with caution due to wide confidence intervals and limited generalizability^35,38^. In contrast, more widely studied interventions—VOT^12,14,15,27,34,36,37,39^, SMS reminder^25,26,29–31,33^, and MERM^17,25,28,32^—consistently demonstrated significant effects with narrower confidence intervals, reinforcing their credibility and replicability across different populations and study settings.

Analysis by country income level further refined our understanding of contextual applicability. In high-income countries, VOT demonstrated statistically significant improvements in adherence, likely supported by stronger digital infrastructure and user literacy^15,36,37,39^. Findings related to IS were inconclusive due to limited data^38^. In upper-middle-income settings, MERM^25,28,32^ and SMS reminder^25,26,29^ emerged as particularly effective, offering a balance between feasibility and impact. In lower-middle- and low-income settings, no included studies showed statistically significant effects of DAT^12,17,31,33^. These findings should be interpreted with caution due to the limited number of trials and possible implementation barriers such as infrastructure, connectivity, and user engagement.

These findings hold significant implications for clinical practice and TB policy. Program planners and health authorities should prioritize DAT solutions that match their local health system capacity and digital infrastructure. For example, SMS reminder may serve as a low-cost entry point in low-resource settings, while video-based approaches may be suitable in urban or high-income areas with adequate broadband and smartphone penetration. Hybrid models, such as the integration of SMS with video check-ins or adherence dashboards, may further enhance flexibility and scalability. Implementing these technologies should be supported by patient education, provider training, and integration with existing TB surveillance systems.

This review is strengthened by its rigorous methodology, including adherence to PRISMA guidelines, the use of a registered protocol, comprehensive multi-database searches, and independent screening and appraisal by multiple reviewers. The exclusive inclusion of RCTs enhances internal validity. Subgroup and sensitivity analyses confirmed the robustness of findings, while Egger’s test (*p* = 0.979) and a slight asymmetry funnel plot suggested minimal risk of publication bias.

Nonetheless, several limitations should be noted. First, substantial heterogeneity was observed across studies, reflecting differences in study design, intervention delivery, population characteristics, and adherence measurement. Although subgroup analyses were performed by DAT type and country income level, heterogeneity remained high in several subgroups (e.g., SMS, MERM), suggesting the influence of other unmeasured or context-specific factors. This justifies the use of a random-effects model, but also indicates that caution is warranted when generalizing pooled estimates across settings. Second, most trials did not implement blinding—understandable given the nature of digital interventions—but this may have introduced performance or detection bias. Third, interventions such as IS^38^ and BMS^35^ were assessed in relatively few studies, limiting the strength of conclusions for those modalities.

Future research should prioritize cost-effectiveness analyses of individual and combined DAT interventions, particularly in low- and lower-middle-income settings where resource allocation is critical. Longitudinal studies are also needed to assess the sustained impact of DATs on treatment completion, relapse prevention, and TB transmission outcomes. In addition, qualitative research exploring patient and provider perspectives could offer insight into digital literacy barriers, acceptability, and real-world feasibility. Evaluations of multi-component or hybrid approaches (e.g., MERM + SMS or VOT + counseling) should be encouraged to determine whether combining modalities enhances adherence more effectively than single interventions alone. Standardizing outcome measures and reporting frameworks would further strengthen comparability and support policy translation across diverse settings.

## Conclusion

This systematic review and meta-analysis demonstrates that DAT such as SMS reminder, VOT, and MERM are generally more effective than conventional DOT, with subgroup analyses supporting their use across multiple contexts. However, some interventions such as IS and BMS were only evaluated in single studies; thus, their consistent superiority over conventional strategies cannot yet be confirmed. Additionally, simplified DATs like SMS reminders may hold promise in lower-income settings, while advanced technologies such as MERM and VOT appear more feasible and effective in upper-middle and high-income countries. These interpretations are based on subgroup findings, but further studies are needed to confirm their implementation suitability across different economic contexts.

## Supplementary information

Appendix A and B

## Data Availability

No datasets were generated or analysed during the current study.
