# Supplementary material for: Digital adherence technology to improve medication adherence in tuberculosis patients: a systematic review and meta-analysis randomized control trials
Source: NPJ Prim Care Respir Med. 2025 Nov 21;35:52. doi: 10.1038/s41533-025-00457-3 (PMC12638299; doi:10.1038/s41533-025-00457-3)
Supplement: Supplementary file 1 — Appendix A and B [file 41533_2025_457_MOESM1_ESM.docx]

**Appendix A: Full Electronic Search Strategies**

**Scopus:**
(Patient* AND Tuberculosis OR TBC OR TB) AND ("Digital AND Adherence* AND Technologi*" OR "Drugs AND Supervisor*" OR DAT OR DOT*) AND (Compliance* OR Adherence* OR "Medication AND Adherence*")

**EBSCO:**
(Patient* AND Tuberculosis OR TBC OR TB) AND ("Digital Adherence Technologi*" OR "Drugs Supervisor*" OR DAT OR DOT*) AND (Compliance* OR Adherence* OR "Medication Adherence*")

**ScienceDirect:**
("Patient Tuberculosis" OR TBC OR TB) AND ("Digital Adherence Technologi" OR "Drugs Supervisor" OR DAT OR DOT) AND ("Medication Compliance" OR Adherence)

**PubMed:**
(Patient* AND Tuberculosis OR TBC OR TB) AND ("Digital AND Adherence* AND Technologi*" OR "Drugs AND Supervisor*" OR DAT OR DOT*) AND (Compliance* OR Adherence* OR "Medication AND Adherence*")

**Appendix B. Detailed Search and Study Selection Process**

**B1. Database Search Results**

A comprehensive search was conducted across four major databases: **Scopus, EBSCO (Medline), ScienceDirect, and PubMed**. The number of records retrieved and filtered at each step is summarized below:

- **Scopus**
  - Records retrieved: 809
  - Articles identified: 644
  - After English language filter: 593
  - Articles eligible for evaluation: 276
- **EBSCO (Medline)**
  - Records retrieved: 639
  - Articles identified: 638
  - After English language filter: 586
  - Articles eligible for evaluation: 211
- **ScienceDirect**
  - Records retrieved: 2,841
  - Articles identified: 1,325
  - After English language filter: 1,312
  - Articles eligible for evaluation: 438
- **PubMed**
  - Records retrieved: 543
  - Articles identified: 62
  - After English language filter: 60
  - Articles eligible for evaluation: 41

**Total records retrieved from all databases: 966**

**B2. Screening and Eligibility Process**

- Duplicate records removed: 67
- Records excluded by automation tools: 105
- Records screened after deduplication: 794
- Records excluded after title/abstract screening: 600
- Full-text articles sought for retrieval: 189
- Evaluating only DAT or DOT without a comparator group: 104
- Full-text articles assessed for eligibility: 85
- Full-text articles excluded:
  - Observational studies: 60
  - Study protocols: 4
  - The Systematic reviews: 2

**B3. Final Inclusion**

- Randomized controlled trials included in final review and meta-analysis: 19

**Notes:**

- Search was limited to articles published in English.
- Articles included were published from inception through November 7, 2024.
